# Polymorphic Variant Associated with Sex Hormone-Binding Globulin Level Is a Risk Factor for Preeclampsia

**DOI:** 10.3390/ijms27188387

**Published:** 2026-09-20

**Authors:** Maria Churnosova, Evgeny Reshetnikov, Inna Sorokina, Inna Aristova, Kirill Tsoy, Mikhail Voronin, Maria Abramova, Alexey Polonikov, Maria Solodilova, Mikhail Churnosov, Irina Ponomarenko

**Affiliations:** 1 Department of Medical Biological Disciplines, Belgorod State National Research University, 308015 Belgorod, Russia; churnosovamary@gmail.com (M.C.); reshetnikov@bsuedu.ru (E.R.); sorokina@bsuedu.ru (I.S.); aristova@bsuedu.ru (I.A.); tsoy@bsuedu.ru (K.T.); 142380@bsuedu.ru (M.V.); abramova_myu@bsuedu.ru (M.A.); polonikov@rambler.ru (A.P.); solodilovama@kursksmu.net (M.S.); ponomarenko_i@bsuedu.ru (I.P.); 2 Department of Biology, Medical Genetics and Ecology, Kursk State Medical University, 305041 Kursk, Russia; 3 Research Institute for Genetic and Molecular Epidemiology, Kursk State Medical University, 305041 Kursk, Russia

**Keywords:** sex hormone-binding globulin, preeclampsia, SNP, association

## Abstract

The present study was devoted to the analysis of associations of single-nucleotide polymorphisms (SNPs) of genes affecting the concentration of circulating sex hormone-binding globulin (SHBG) (information was obtained from genome-wide association studies [GWAS]) with the development of preeclampsia (PE). This retrospective study was performed with a case–control design using a sample of 891 pregnant women, including women with PE [n = 431] and without PE (control) [n = 460] who underwent an experimental genetic study of 11 SNPs (rs17496332 [*PRMT6*], rs780093 [*GCKR*], rs10454142 [*PPP1R21/KLRAQ1*], rs3779195 [*BAIAP2L1*], rs440837 [*ZBTB10*], rs7910927 [*JMJD1C*], rs4149056 [*SLCO1B1*], rs8023580 [*NR2F2-AS1*], rs12150660 [*SHBG*], rs727428 [*SHBG*], and rs1641549 [*TP53*]) that had previously shown a connection with the concentration of circulating SHBG in GWAS. As a result of the associative analysis, it was revealed that the polymorphism rs10454142 *PPP1R21/KLRAQ1* was associated with PE risk [the data were obtained within the framework of a recessive genetic model]: the CC genotype of this SNP had an impact risk value for PE (OR: 1.73; 95%CI: 1.16–2.66; *p*_perm_: 0.014). The PE-associated variant rs10454142 *PPP1R21/KLRAQ1* and its proxy SNPs demonstrate potential pronounced functionality both in the liver (the main organ of SHBG formation) and organs/cell cultures targeted for PE (trophoblast, amnion, placenta, and uterus), thereby affecting the regulation of gene transcription, nucleic acid metabolism, and embryo development. In conclusion, this exploratory study was the first to show the risk effect of the SHBG-related genetic variant in the formation of PE.

## 1. Introduction

Preeclampsia (PE) is one of the most common complications of pregnancy (its frequency varies on average between 2–8% in different populations [1]). PE manifests after the 20th week of pregnancy with symptoms such as hypertension, proteinuria, and often dysfunction of various organs (kidneys, brain, liver, blood, etc.) [2,3,4]. PE has serious consequences for both the mother and the fetus, and these consequences can often be fatal. Every year, PE causes over 75,000 maternal deaths and 500,000 intrauterine/neonatal deaths worldwide [5]. Women who have undergone PE have a higher risk of developing cardiovascular diseases (hypertension, etc.) and their complications (stroke, etc.) as well as diabetes mellitus, resulting in a low life expectancy [3,4]. Children born as a result of pregnancy complicated by PE have an increased risk of the nervous and cardiovascular systems diseases and metabolic disorders during life [3,4].

Risk factors (genetic, age of the woman, obesity, hormonal disorders, etc.) and mechanisms of development (pathological placentation, endothelial dysfunction, oxidative stress, systemic inflammation, etc.) of PE are actively studied by various scientific teams [1,2,3,4,6,7,8,9,10,11,12,13,14,15,16,17,18,19,20,21]. To date, convincing data have been obtained on the significant role of genetic factors in the formation of PE (31–54%) [22,23,24] with a predominant contribution (64–70% of all genetic factors) to the pathogenetics of this pregnancy complication in the maternal genome [25,26]. The currently available results of genome-wide association studies (GWAS) of PE only partially “reveal” the role of individual polymorphisms of candidate genes in the pathophysiology of PE (for example, *PLEKHG1* [rs9478812], *INHBB* [rs7579169], *ZNF831* [rs259983], *FTO* [rs1421085], etc. [26,27,28,29,30]); due to the limited evidence obtained, they do not provide a complete “picture” of the genetic nature of this pregnancy complication.

The onset of pregnancy and its progression are accompanied by significant hormonal changes in the female body, and this primarily concerns sex hormones [1,31]. An imbalance in these hormonal profile rearrangements can cause abnormalities during the normal course of pregnancy and contribute to the development of complications (including PE) [1,32,33,34]. The most important “regulator” of the level/activity of sex hormones (androgens/estrogens) in the body is sex hormone-binding globulin (SHBG) [35,36]. This protein, by binding sex hormones (65% of testosterone and 38% of estradiol are bound to SHBG), directly determines the concentrations of their free fractions (they account for only 1–2% of the total amount of androgens/estrogens in the body), which are bioactive and realize phenotypic effects in the body [37,38]. Thus, the concentration of circulating SHBG in the organism, which is largely genetically determined (there are GWAS data on numerous genetic determinants of SHBG [39,40,41,42,43,44,45,46,47,48,49,50,51,52,53,54,55]), directly affects the level of biologically active androgens/estrogens in the body, which may be important for the pathophysiology of PE [32,33,34,56,57,58,59]. There is evidence in the literature (obtained by the MR GWAS method from the materials presented in the FinnGen and UK Biobank databases) of a negative causal relationship (OR = 0.823–0.917) of SHBG with various disorders of pregnancy (gestational hypertension/diabetes, bleeding in early pregnancy, miscarriage, premature birth, etc.) that may indicate the potential “serious” involvement of SHBG in the pathogenetics of obstetric complications [59]. Further genetic studies are needed to detect/confirm the association of SHBG (its genetic determinants) with the risk of PE [57]. The aim of our study was to analyze the associations of SHBG single-nucleotide polymorphisms (SNPs) with the development of PE using GWAS.

## 2. Results

The detailed phenotypic characteristics of the study participants (including the clinical characteristics of the current pregnancy) are presented in Table 1. In the group of women with PE, early onset of PE was observed in 14.15%, severe PE was diagnosed in 16.70%, and fetal growth restriction was identified in 19.26%.

### 2.1. Analysis of Associations Between SHBG-Related Genetic Variants and PE

As a result of the associative analysis, it was revealed that polymorphism rs10454142 *PPP1R21/KLRAQ1* was associated with the PE risk [the data were obtained within the framework of a recessive genetic model]: the CC genotype of this SNP had an impact risk value for PE (OR: 1.73; 95% CI: 1.16–2.66; *p*: 0.015; *p*_perm_: 0.014; 1434 permutations were performed, power_post-hoc_: 77.57%) (Table 2).

### 2.2. Replication of Associations of rs10454142 PPP1R21/KLRAQ1 and Its Proxy SNPs with PE Using GWAS Data (UK Biobank and FinnGen Database)

**Figure 1:**
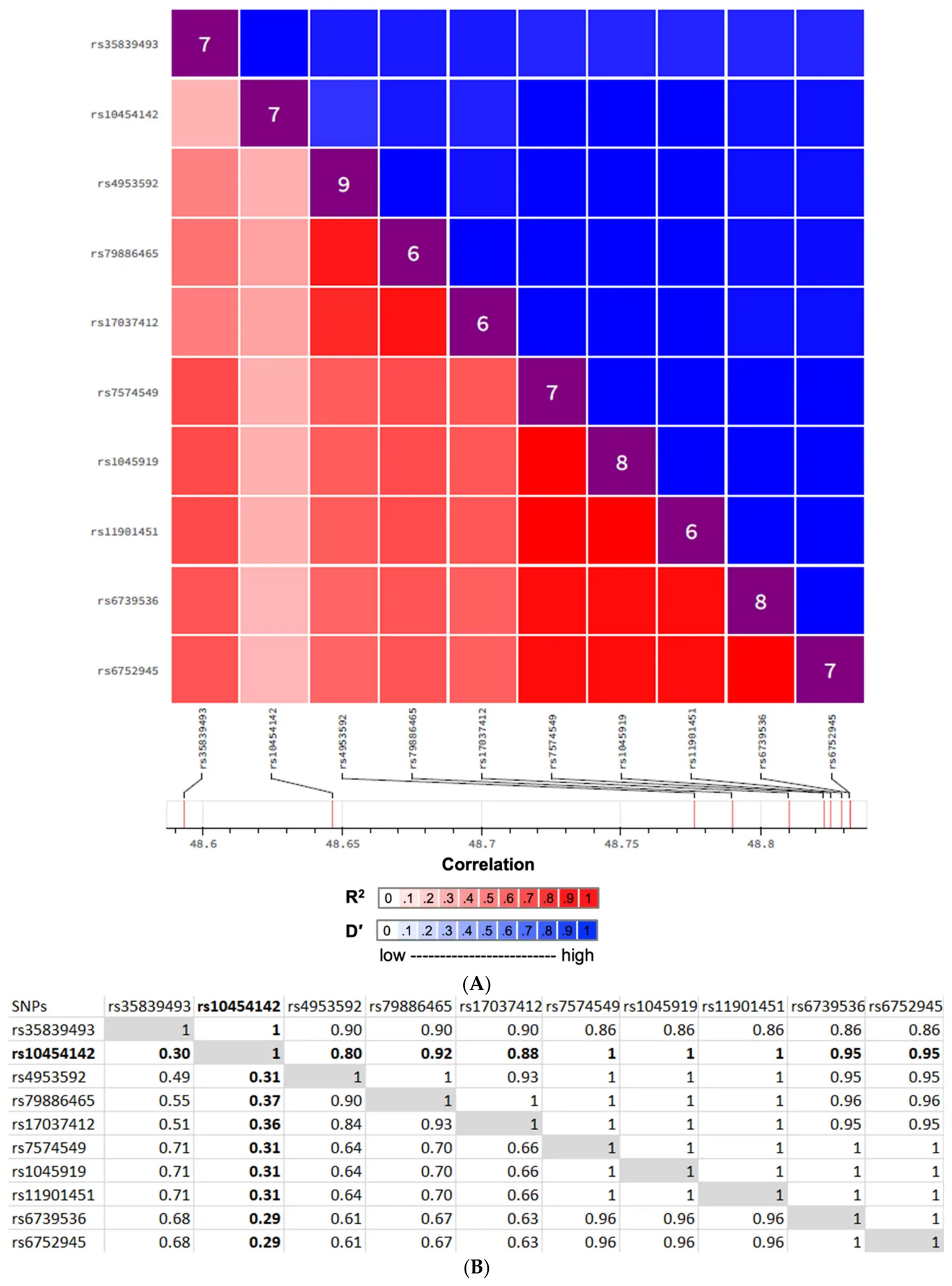
Indicators of LD (r^2^ and D′) between the PE-correlated SNP rs10454142 *PPP1R21/KLRAQ1* (our data) and its proxy variants (SNPs are ordered by genomic coordinates) associated with PE according to UK Biobank and FinnGen GWAS data: interactive heat map (**A**); matrix of pairwise LD statistics (**B**) (r^2^, bottom left; D′, top right) (the data were obtained using the LDlink program among Europeans (the 1000 Genomes Project population—Finnish in Finland (FIN))); the data for the PE-associated locus SNP rs10454142 *PPP1R21/KLRAQ1* are highlighted in bold.

As a result of a replicative analysis of the associations of rs10454142 *PPP1R21/KLRAQ1* and its proxy SNPs with PE using available GWAS data (we analyzed such phenotypes as “gestational hypertension/preeclampsia” (UK Biobank GWAS data) and “preeclampsia” (FinnGen GWAS data, meta-analysis of UK Biobank and FinnGen GWAS data)), the following results were obtained (Table 3). Unfortunately, rs10454142 *PPP1R21/KLRAQ1* did not confirm the association with PE according to GWAS data from the UK Biobank and the FinnGen database (*p* > 0.05). At the same time, several polymorphisms (9 SNPs) that were in LD with rs10454142 *PPP1R21/KLRAQ1* (0.29 ≤ r^2^ ≤ 0.37 and 0.80 ≤ D′ ≤ 1.00 (Figure 1)) showed significant associations (0.008 ≤ *p* ≤ 0.050) with the “gestational hypertension/preeclampsia” phenotype (UK Biobank GWAS data) (all 9 loci). Thus, it is associated with the “preeclampsia” phenotype (FinnGen GWAS data, meta-analysis of UK Biobank and FinnGen GWAS data) (four loci) (Table 3). It should be noted that these nine above-mentioned variants are not only proxy variants of rs10454142 *PPP1R21/KLRAQ1* but are also in LD between themselves (0.49 ≤ r^2^ ≤ 1.00 and 0.86 ≤ D′ ≤ 1.00) (Figure 1). Thus, as a result of the replication, although we have not received direct evidence of the association of rs10454142 *PPP1R21/KLRAQ1* with PE, the identified associations of several of its proxy variants with PE may support our assumption of the possible involvement of this genome region (chromosome 2:48593077-48831931 (GRCh37)) in the formation of PE. Definitely, additional associative and replicative genetic studies on this topic are needed.

### 2.3. Predicted Functionality of the PE-Associated Locus rs10454142 PPP1R21/KLRAQ1 and Its Proxy SNPs

As a result of the analysis of the potential functionality of the PE-associated locus rs10454142 *PPP1R21/KLRAQ1* and its proxy SNPs, the following data were obtained (Table 4 and Tables S1–S4).

First, the genome region we are considering was functionally important in the liver: (a) three polymorphisms (the PE-associated SNP rs10454142 *PPP1R21/KLRAQ1* and two of its proxy variants, rs10454143 and rs201414717) of the 11 SNPs under consideration were localized in the region of potential promoters/active promoters [labeled by such modified (methylation/acetylation) proteins and histones as H3K4me3/H3K9ac] and enhancers/active enhancers [H3K4me1/H3K27ac] in the region of the *PPP1R21* and *KLRAQ1* genes (Table 4 and Table S1); (b) the PE-associated polymorphism rs10454142 *PPP1R21/KLRAQ1* and 5 variants in LD with it were associated with the expression (eQTL) of the *GTF2A1L* gene (Table 4 and Table S2); (c) nine of the 11 studied SNPs (rs10454142 *PPP1R21/KLRAQ1* and eight strongly coupled variants) were correlated with alternative splicing (sQTL) of the *PPP1R21* gene (Table 4 and Table S3); (d) the PE-associated SNP rs10454142 *PPP1R21/KLRAQ1* and nine of its proxy variants were involved in the regulation of DNA methylation (mQTL) in the region of the *FOXN2* gene [cg00032884 (chr2:48540671), cg15846641 (chr2:48541264)] (Table 4 and Table S4). Interestingly, in the liver, the PE risk allele C rs10454142 *PPP1R21/KLRAQ1* (OR = 1.74) was associated with DNA hypermethylation in the region of the *FOXN2* gene (β = 0.46; *p* = 4.3 × 10^−9^) (Table S5), low eQTL of the *GTF2A1L* gene (NES = −0.47; *p* = 1.2 × 10^−6^) (Table S2), and a multidirectional effect (increase/decrease) on the sQTL in the region of two introns [IntronID:48505596:48507269:clu_28930_+ (NES = 0.53; *p* = 4.9 × 10^−9^) and IntronID: 48505857:48507269:clu_28930_+ (NES = −0.45; *p* = 2.8 × 10^−7^)] of the *PPP1R21* gene (Table S3).

Second, rs10454142 *PPP1R21/KLRAQ1* and strongly linked variants exhibit pronounced functionality in PE-targeted organs/cell cultures (Table 4 and Tables S1–S5): (a) in cultured trophoblast cells (H1 BMP4-derived trophoblast cultured cells; Mnemonic:ESDR.H1.BMP4.TROP;EpigenomeID:E005) five of the 11 studied loci (including the PE-associated SNP rs10454142 *PPP1R21/KLRAQ1*) were located in the region of potential enhancers (H3K4me1) (Table 4 and Table S1); (b) in the amnion (placenta amnion; EpigenomeID: E099; Mnemonic: PLCNT.AMN), three polymorphisms (the PE-associated SNP rs10454142 *PPP1R21/KLRAQ1* and its two LD variants, rs201414717 and rs10454143) were localized in the region of putative enhancers [H3K4me1] and active enhancers [H3K27ac] in the *PPP1R21* and *KLRAQ1* genes (Table 4); (c) in the placenta (placenta; Mnemonic: PLCNT.FET; EpigenomeID: E091), the three above-mentioned SNPs are located in regions of the genome where assumed active enhancers [H3K27ac] were presented in the region of the *PPP1R21* and *KLRAQ1* genes (Table 4); (d) in the uterus, nine polymorphisms (including the PE-associated locus rs10454142 *PPP1R21/KLRAQ1* and eight of its proxy SNPs) were associated with the sQTL of the *PPP1R21* gene (Table 4). Interestingly, the PE risk allele C rs10454142 *PPP1R21/KLRAQ1* (OR = 1.74) was correlated with a low sQTL level of two intronic regions of the *PPP1R21* gene [IntronID: 48505857:48507269:clu_30732_+ (NES = −0.47; *p* = 2.7 × 10^−8^) and IntronID: 48505596:48505718:clu_30732_+ (NES =−0.49; *p* = 1.5 × 10^−9^)] in the uterus (Table S3).

Third, the PE-associated SNP rs10454142 *PPP1R21/KLRAQ1* and its proxy variants were functionally significant in organs/tissues important for the pathophysiology of PE: the brain, including the cortex (eQTL:*STON1*, *PPP1R21*, *PPP1R21-DT*; sQTL:*PPP1R21*; mQTL:*FOXN2*), pituitary gland (eQTL:*PPP1R21*, *FOXN2*, *GTF2A1L*, *STON1-GTF2A1L*; sQTL:*PPP1R21*), and hypothalamus (eQTL:*GTF2A1L*, *PPP1R21*; sQTL:*PPP1R21*); adipose tissue (eQTL:*MIR548BAHG*, *STON1*, *FOXN2*, *PPP1R21*, *STON1-GTF2A1L*, *GTF2A1L*; sQTL:*STON1*, *PPP1R21*, *STON1-GTF2A1L*); ovaries (sQTL:*PPP1R21*); thyroid gland (eQTL:*GTF2A1L*, *STON1*, *PPP1R21*, *FOXN2*; sQTL:*PPP1R21*; 3′aQTL:*PPP1R21*); blood (mQTL:*FOXN2*, *PPP1R21*, *KLRAQ1*); arteries (eQTL:*MIR548BAHG*, *GTF2A1L*, *STON1-GTF2A1L*, *STON1*, *LHCGR*, *PPP1R21*; sQTL:*STON1*, *PPP1R21*, *STON1-GTF2A1L*); and adrenal glands (eQTL:*GTF2A1L*, *FOXN2*; sQTL:*PPP1R21*). The associations of the PE risk allele C rs10454142 *PPP1R21/KLRAQ1* with the eQTL/sQTL/mQTL levels of various genes were multidirectional. For example, in the pituitary gland, this allele was linked with ↑eQTL *PPP1R21* (NES = −0.21; *p* = 4.5 × 10^−11^) as well as ↓eQTL *FOXN2* (NES = −0.28; *p* = 6.4 × 10^−6^) and *GTF2A1L* (NES = −0.45; *p* = 2.2 × 10^−11^); Similarly, in the thyroid gland, the allele C rs10454142 was correlated with ↑eQTL *STON1* (NES = 0.13; *p* = 1.0 × 10^−5^) and *PPP1R21* (NES = 0.77; *p* = 3.0 × 10^−111^) as well as ↓eQTL *GTF2A1L* (NES = −0.24; *p* = 1.6 × 10^−14^) (Table S2).

Fourth, in the body as a whole, rs10454142 *PPP1R21/KLRAQ1* and 10 of its LD variants demonstrate significant functionality (Table 4 and Tables S1–S5). They were involved in the following: (a) the interaction of a genome region in the area of the *PPP1R21/KLRAQ1/FOXN2* genes with more than 50 transcription factors (TFs) (n = 51: ZNF219, Zfp105, ZBRK1, YY1, WT1, TCF4, TCF12, TAL1, STAT, Sox, RREB-1, Rad21, PRDM1, Pax-4, p300, NF-kappaB, NF-E2, Myc, MIZF, Maf, LBP-1, KAP1, Irf, Hoxb6, Foxa, Fox, FAC1, CIZ, ELF1, CEBPD, CACD, BCL, BATF, Barx1, BAF155, Bach2, Bach1, AP-4, AP-2, AP-1, Hoxa5, GR, Hoxa3, HMGN3, HDAC2, GATA, Foxp1, Foxo, Foxk1, Foxj2, Foxd3) (Table 4). Nine of the 11 polymorphisms under consideration (81.82%, including the PE-associated SNP rs10454142 *PPP1R21/KLRAQ1*) play a significant role in TF–DNA interactions (Table 4). It is important to note that these nine SNPs exhibit TF-related effects both in the body as a whole, in the liver, and in organs/cell cultures targeted for PE (trophoblast, amnion, placenta, uterus). Interestingly, the PE risk allele C rs10454142 *PPP1R21/KLRAQ1* significantly increases affinity (LOD_scores_ = +7.8) to TF NF-kappaB; (b) eQTL of the 11 genes (9/11 SNPs [81.82%]: *ELOBP3*, *MIR548BAHG*, *PPP1R21-DT*, *STON1-GTF2A1L*, *FOXN2*, *FSHR*, *GTF2A1L*, *LHCGR*, *MSH6*, *PPP1R21*, *STON1*) (Table 4 and Table S2); (c) sQTL of the four genes (9/11 SNPs [81.82%]: *FOXN2*, *PPP1R21*, *STON1*, *STON1-GTF2A1L*) (Table 4 and Table S3); (d) alternative polyadenylation (3′aQTL) of the *PPP1R21* gene (9/11 SNPs [81.82%]) (Table 4 and Table S4); and (e) mQTL of the three genes (11/11 SNPs [100.00%]: *FOXN2*, *PPP1R21*, *KLRAQ1*) (Table 4 and Table S5). Interestingly, the PE risk allele C rs10454142 *PPP1R21/KLRAQ1* was multidirectionally associated with eQTL/sQTL/mQTL of various genes.

### 2.4. Identification of Possible PE-Related Biological Pathways

**Figure 2:**
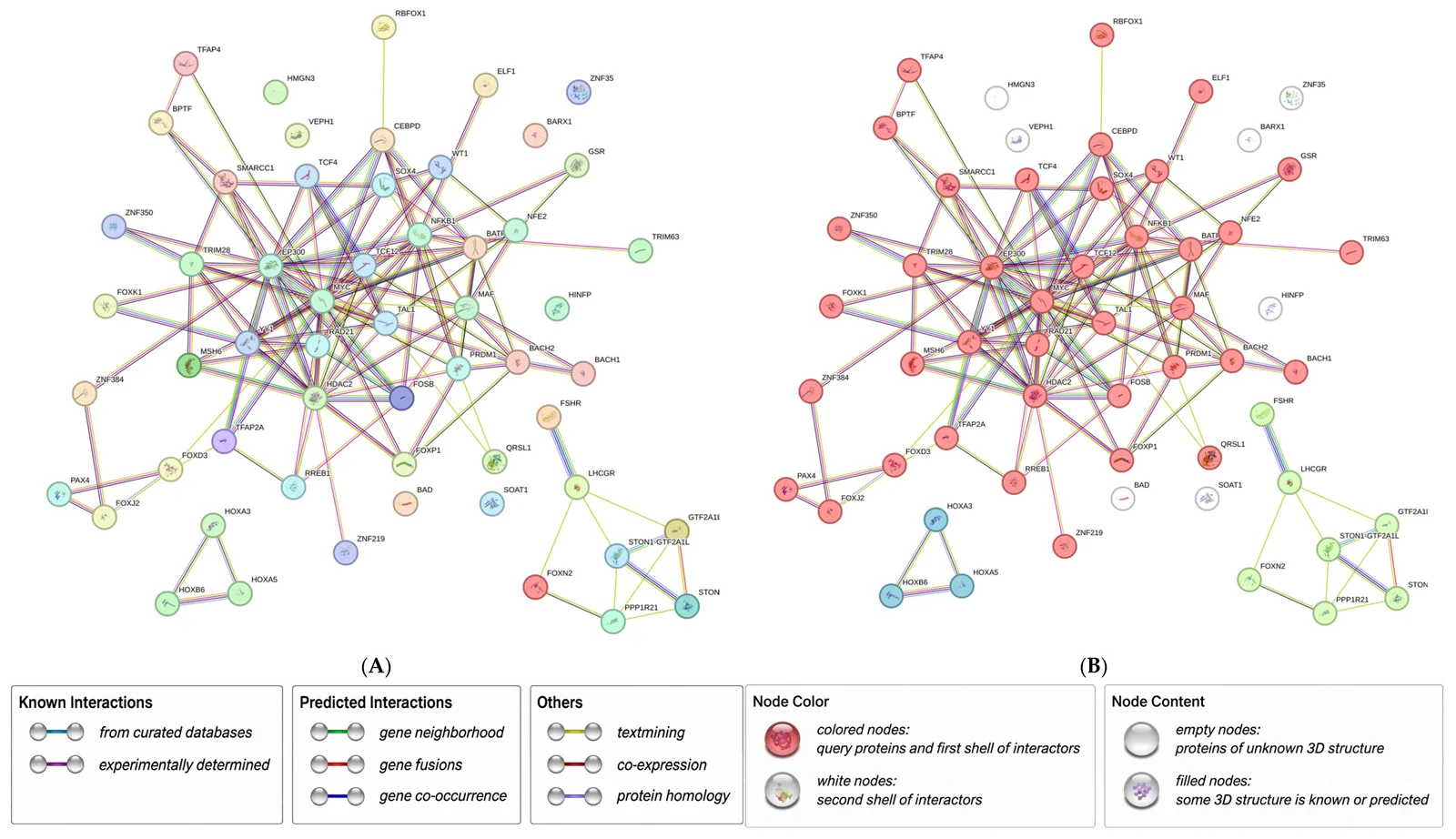
Interaction network of PE-correlated transcription factors and proteins mediated by rs10454142 *PPP1R21/KLRAQ1* and proxy variants: the resulting network (**A**) and its clustering (**B**) (cluster 1 [indicated in red], cluster 2 [indicated in green], and cluster 3 [indicated in blue]) (STRING data).

At the final stage of our study, using the above data on the functionality of the PE-associated locus rs10454142 *PPP1R21/KLRAQ1* and 10 SNPs strongly linked to it (r^2^ ≥ 0.80), we evaluated the putative pathways involving genes/proteins/TFs “controlled” by these genetic variants. Here, we studied the biological pathways involving 12 genes and 51 TFs functionally related (epigenetic effects/eQTL/sQTL/3′aQTL/mQTL) to rs10454142 *PPP1R21/KLRAQ1* and its proxy SNPs (r^2^ ≥ 0.80) (Table 4). The network of important PE protein–protein interactions (P-P) of TFs/proteins mediated by rs10454142 *PPP1R21/KLRAQ1* (and its proxy variants) and its clustering (three clusters in total) are shown in Figure 2. The first P-P cluster (the most numerous, comprising 39 TFs (Figure 2B)) was involved in a variety of biological processes (about 150) (Table S6) related to regulation: (a) gene transcription (positive/negative regulation of transcription by RNA polymerase II, gene expression, chromatin remodeling, regulation of miRNA transcription, estrogen-dependent gene expression, etc.); (b) metabolic processes of nucleic acids (regulation of nucleobase-containing compound metabolic process, DNA, RNA and miRNA metabolism process, etc.); (c) embryo development (regulation of cell differentiation, embryonic development, immune system and kidney development, regulation of heart growth, etc.).

The second P-P cluster, comprising seven proteins/TFs (GTF2A1L, FOXN2, LHCGR, FSHR, PPP1R21, STON1-GTF2A1L, and STON1) (Figure 2B) was mainly involved in the pathways associated with the transcription factor TFIIA complex (GO:0005672; *p*FDR = 0.0062 [STON1-GTF2A1L, GTF2A1L]), ovarian steroidogenesis (hsa04913; *p*FDR = 0.0478 [LHCGR, FSHR]), and hormone ligand-binding receptors (HSA-375281; *p*FDR = 0.0224 [LHCGR, FSHR]).

The three TFs (HOXA3, HOXA5, HOXB6) were included in the third P-P cluster (Figure 2B) that belongs to homeobox proteins and are part of the developmental regulatory system that provides cells with a specific location on the anterior-posterior axis. They were involved in the processes of bone morphogenesis in embryogenesis (embryonic skeletal system morphogenesis 3 (GO:0048704, pFDR = 0.0019 [HOXA5, HOXB6, HOXA3]); anterior/posterior pattern specification (GO:0009952, pFDR = 0.0071 [HOXA5, HOXB6, HOXA3]); and thyroid gland development (GO:0030878, pFDR = 0.0151 [HOXA5, HOXA3])).

## 3. Discussion

This exploratory study is the first to show the correlation between an SHBG-related genetic variant and the formation of PE: the CC genotype of rs10454142 *PPP1R21/KLRAQ1* had a risk value for PE (OR:1.73). The associations of several proxy variants rs10454142 *PPP1R21/KLRAQ1* with PE revealed by us in a replicative study may support our assumption that this region of the genome (chromosome 2:48593077-48831931 (GRCh37)) may be involved in the formation of PE. The PE-associated variant rs10454142 *PPP1R21/KLRAQ1* and its proxy SNPs demonstrate potentially pronounced functionality both in the liver (the main organ of SHBG formation) and in organs/cell cultures targeted for PE (trophoblast, amnion, placenta, uterus), thus affecting the regulation of gene transcription, nucleic acid metabolism, and embryo development.

Initially, GWAS data on the association of the PE-asrs10454142 *PPP1R21/KLRAQ1* locus considered in this work with concentration of circulating SHBG in the body were obtained from the work of Coviello et al. [40]. According to the data of this team of authors, the reference (frequent) allele T rs10454142 *PPP1R21/KLRAQ1* was correlated with a high concentration of circulating SHBG (β = 0.026), and, accordingly, the alternative (rare) allele C was associated with a low content of SHBG [40]. Thus, taking into account our results, we can assume that if the SHBG-lowering genetic variant—genotype CC of the rs10454142 *PPP1R21/KLRAQ1*—is present in the genotype of a woman, it may increase (by 73%) the risk of PE (OR: 1.73).

The rs10454142 *PPP1R21/KLRAQ1* polymorphism (and a number of its proxy SNPs), according to our in silico data, have pronounced putative functionality in the liver, where SHBG is predominantly formed [36]. They are localized in the region of potential promoters/active promoters and enhancers/active enhancers in the areas of the *PPP1R21* and *KLRAQ1* genes. They are associated with the expression of the *GTF2A1L* gene and alternative splicing of the *PPP1R21* gene and are involved in regulating the level of DNA methylation in the region of the *FOXN2* gene. At the same time, in the liver, the PE risk allele C rs10454142 *PPP1R21/KLRAQ1* (genotype CC, OR: 1.73) was associated with DNA hypermethylation in the region of the *FOXN2* gene (β = 0.46), low eQTL of the *GTF2A1L* gene (NES = −0.47), and a multidirectional effect (increase/decrease) on the sQTL of the *PPP1R21* gene in the region of two introns [IntronID: 48505596:48507269:clu_28930_+ (NES = 0.53) and IntronID: 48505857:48507269:clu_28930_+ (NES = −0.45)]. This may be the biomedical basis for its involvement in the regulation of SHBG formation in the liver. Significant phenotypic effects in the liver (association with the level of individual liver enzymes) have been reported in a number of GWAS and for several proxy variants rs10454142 *PPP1R21/KLRAQ1*, such as rs10208627 (alanine aminotransferase [9], r^2^ = 0.56/D′ = 1.00), rs6749773 (alkaline phosphatase [9,60,61], r^2^ = 0.53/D′ = 1.00), and rs13429377 (gamma-glutamyltranspeptidase [9], r^2^ = 0.59/D′ = 1.00), which also confirms the important role of the genome region in the rs10454142 *PPP1R21/KLRAQ1* area in the regulation of liver functional activity.

A number of publications have been devoted to the issue of the relationship between concentration of circulating SHBG and PE [34,35,56,57,58,59,62,63]. The literature indicates several mechanisms explaining the relationship between SHBG and hypertensive disorders of pregnancy (PE/gestational hypertension) [34,35,58,62,63,64,65]. First, low levels of SHBG were associated with metabolic disorders, such as insulin resistance, systemic inflammation, etc., which exacerbate vascular dysfunction and oxidative stress. These have been reported as key factors of PE, placental insufficiency, and gestational hypertension [62,63,65]. Using the Mendelian randomization method and GWAS data provided in the databases of FinnGen, UK Biobank, and Gan et al., a negative causal relationship was found between SHBG and pregnancy disorders such as gestational hypertension (OR = 0.917), gestational diabetes (OR = 0.835), and bleeding in early pregnancy (OR = 0.853) [59]. Second, SHBG can affect angiogenic factors that are crucial for the development of the placenta; however, disorders in this process lead to disruption of utero-placental blood flow and the development of PE [34]. Third, SHBG regulates the balance of sex hormones, namely, androgens/estrogens, in the body, directly determining the level of their active (bioavailable) forms [35,36,66]. It should be noted that in pregnant women with PE, testosterone levels were significantly increased (differences with controls reach 2–3 times) [32]. There is observational evidence indicating a high level of estradiol in pregnant women with PE in early pregnancy (1.5 times higher than the same indicator in the control group) [67,68].

Thus, the risk of the SHBG-lowering genetic variant of the CC genotype rs10454142 *PPP1R21/KLRAQ1* established in our work during the formation of PE (OR: 1.73) fully corresponds to the above data on the biological role of SHBG in the body and thus has a serious medical and biological justification.

The results of previous studies on the association of SHBG with PE are ambiguous and contradictory [56,57,58,59]. On the one hand, the literature presents the results of three studies performed using the Mendelian randomization method of the same GWAS data obtained from the FinnGen and UK Biobank databases, in which the authors did not find a causal relationship between SHBG and the development of PE [56,57,58,59]. At the same time, the authors conclude that the results of their research contradict numerous previously performed observational studies in this area and indicate the need for additional research on this topic [57]. Thus, there is an obvious need to obtain new GWAS data on this topic in new elections, rather than multiple “re-analysis” of existing GWAS materials, which will not allow us to obtain new knowledge/information on the issue of SHBG–PE communication. On the other hand, completely different conclusions about the association of SHBG with PE are drawn by the authors Malvi et al., who conducted a detailed and meticulous systematic review and meta-analysis on this topic [58]. In their paper, the authors searched the PubMed, Embase, and Web of Science databases for all studies evaluating the relationship between SHBG and the risk of PE/gestational hypertension, and eight studies were included in the review out of a total of 592 articles found (they provided quantitative estimates of the risk of PE/gestational hypertension for SHBG), on the basis of which a meta-analysis was performed. As a result of the meta-analysis, an association between SHBG and the risk of developing any of the considered hypertensive disorders of pregnancy (PE/gestational hypertension—OR = 0.875 (95% CI: 0.772–0.993)) was established. Based on the data obtained, the authors conclude that there is a potentially significant inverse relationship between SHBG and the risk of hypertensive pregnancy disorders (PE/gestational hypertension) and point to the need for further research on this issue to determine the potential usefulness of SHBG as a biomarker for predicting hypertensive pregnancy disorders.

This research paper has a number of limitations, which include the following: (a) the results obtained need additional confirmation (replication) in other territorial groups of Europeans (including Russia), as well as in other ethnic cohorts, and are therefore preliminary; (b) the study did not examine the concentration of SHBG in the blood of the subjects, and the data on the estimated level of SHBG (high/low) in carriers of certain genetic variants were taken from previously conducted GWAS; and (c) the potential functional effects of the PE-associated locus rs10454142 *PPP1R21/KLRAQ1* and its strongly linked SNPs (epiQTL, eQTL, sQTL, mQTL, 3′aQTL) identified in silico in the study, which may be the biological basis for their association with PE, require confirmation in experimental studies.

## 4. Materials and Methods

### 4.1. Study Subjects

When planning this study, we calculated the number of participants (PE/control) that we need to include in the study to obtain representative results (power = 80%, α = 0.05, OR = 1.24–1.52). As a result of the calculations performed (the Quanto program was used (v.1.2.4)), it was determined that the total number of participants should be ≥800 pregnant women.

**Figure 3:**
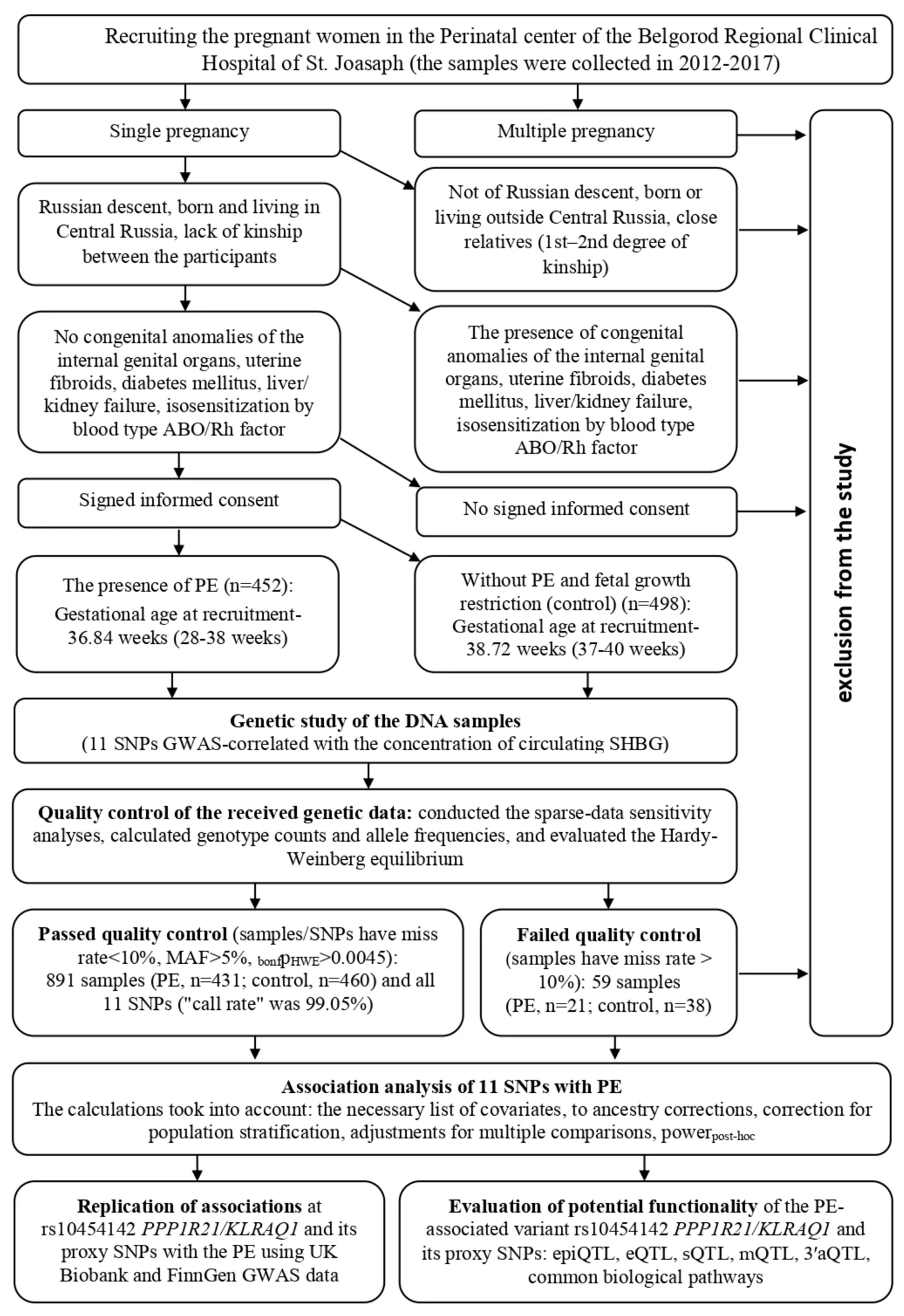
The general outline of the investigation.

This retrospective study was performed as a case–control design on a sample of 891 pregnant women—women with PE [n = 431] and without PE (control) [n = 460]. The general outline of the study is presented in Figure 3. The samples were collected in 2012–2017 in specialized departments at the perinatal center of the Belgorod Regional Clinical Hospital of St. Joasaph. The study included women (a) with a single pregnancy; (b) gestation period 37–40 weeks for women without PE (gestational age at delivery); women with PE were included in the group of participants during hospitalization for treatment/delivery of PE (28–38 weeks); (c) Russian ethnic group (according to the self-identification of women); (d) born and living in Central Russia [69,70]; and (e) lack of kinship between the participants. Women with any of the following diseases were excluded from the study: congenital anomalies of the internal genital organs, uterine fibroids, diabetes mellitus, liver or kidney failure, or isosensitization by blood type Rh factor or ABO [69,70,71,72]. PE was diagnosed by certified obstetricians based on the modern diagnostic criteria [73]: the presence of hypertension in a pregnant woman (systolic blood pressure (BP) 140 mmHg and above and/or diastolic BP 90 mmHg and above), which occurred after 20 weeks of pregnancy (with previously normal blood pressure) combined with either proteinuria (the total protein content/excretion in the urine [within 24 h] was not less than 300 mg) or utero-placental dysfunction or evidence of other maternal end-organ dysfunction. Women without PE and fetal growth restriction were included in the comparison group (control group). The study was carried out with the support of the Medical Ethics Committees of the Belgorod Regional Clinical Hospital of St. Joasaph and Belgorod State University, with the mandatory written informed consent from each subject.

### 4.2. DNA Study: Extraction and SNP Selection/Detection

DNA samples extracted from the venous blood of pregnant women (PE/without PE) (using the ethanol/chloroform/phenolic method [74]) were used for the present genetic study. Laboratory kelvinators were used for DNA storage [75].

The 11 polymorphisms were specially selected for this study based on the following criteria: (1) associations with the concentration of circulating SHBG in previously performed GWAS [40,41,42,43,45,50] (Table 5); (2) association with SHBG in samples of women (mostly women); (3) the presence of GWAS-confirmed associations among Europeans; (4) the presence of the alleged functionality of this loci [76,77,78,79,80,81,82] (estimated on the basis of HaploReg data (accessed 13 December 2025)) [83]) (Table S7); (5) the minor allele frequency among Europeans is >5% (HaploReg data (accessed 13 December 2025)) (Table S7); and (6) the correlations of these loci (GWAS data [43,45,50]) with the content of testosterone and estradiol was also taken into account (Table 5) due to the fact that SHBG is directly involved in regulating the levels of bioavailable (active) testosterone/estradiol [37,38] (Table 5). These were SNPs such as rs17496332 [*PRMT6*], rs780093 [*GCKR*], rs10454142 [*PPP1R21/KLRAQ1*], rs3779195 [*BAIAP2L1*], rs440837 [*ZBTB10*], rs7910927 [*JMJD1C*], rs4149056 [*SLCO1B1*], rs8023580 [*NR2F2-AS1*], rs12150660 [*SHBG*], rs727428 [*SHBG*], and rs1641549 [*TP53*] (Table 5 and Table S7). Detailed information on the 11 loci included in this study (GWAS source, phenotype (SHBG/testosterone/estradiol), effect allele and its effect size) is presented in Table 5. The SNP laboratory testing was performed on a CFX96 device [84]. The quality control of the obtained genetic data was carried out using positive and negative control samples, as well as repeated “blind” genotyping of randomly selected every fifteenth and twentieth DNA sample [85]).

### 4.3. Statistical Analysis

The reliability of differences between the compared groups (PE/control) according to the analyzed phenotypic characteristics (Table 1) was determined using the Mann–Whitney (quantitative signs) and chi-square (qualitative [binary] signs) tests. The differences between the analyzed parameters at *p* ≤ 0.05 were considered statistically significant.

Prior to the association analysis, we conducted the sparse-data sensitivity analyses, calculated genotype counts and allele frequencies, and evaluated the Hardy–Weinberg equilibrium (HW_eq_).

When assessing the sparse-data sensitivity, we excluded samples/SNPs from the analysis that had a miss rate > 10%. Thus, after filtering and excluding a number of samples (n = 59) from the analysis, 891 samples (from the initially analyzed 950 samples) and all 11 SNPs were included in the final genetic database that we used for further calculations. The total genotyping rate (the “call rate” indicator) in this samples/SNPs was 99.05%, which indicates the sufficient quality of the obtained genetic data and the possibility of their use in the statistical analysis of associations. The data obtained on the genotype counts and frequencies of minor alleles (MAFs) in the studied groups of women (PE and PE free) are presented in Table S8. All the studied SNPs are quite polymorphic both in the PE (MAF ≥ 0.180) and in the PE free (MAF ≥ 0.209) cohorts. We evaluated the Hardy–Weinberg equilibrium (HW_eq_) for all 11 loci in the PE/without PE groups to additionally verify the quality of the experimental data obtained [86]. When checking the correspondence in the genotype distribution (actual/expected) for the 11 loci under consideration, no statistically significant deviations from HWE were found in PE (0.173 < *p*_HWeq_ < 0.772) and PE free (0.038 < *p*_HWeq_ < 1.000) (Table S8) women (the analysis was performed taking into account the additionally introduced Bonferroni correction equal to the number of studied polymorphisms [n = 11], _bonf_*p*_HWE_ > 0.0045 [0.05/11]).

The associations between SNPs and PE was assessed using regression analysis (Firth’s penalized regression) based on ORs (odds ratios) and 95% CIs (confidence interval) OR [87,88] for three generally accepted (additive, dominant, recessive) genetic models [89,90] in the gPlink program [Java-adapted version] (PLINK v2.0.0-a.7.1) [91]. The calculations took into account the following:

(a) The necessary list of covariates (age and BMI/obesity before pregnancy, family history of PE, number of pregnancies/abortions/stillbirths (materials from Table 1));
(b) Ancestry corrections in the studied sample; a genomic relationship matrix (GRM) based on the identity-by-similarity (IBS) was built and then taken into account when calculating the principal components;
(c) The first principal component (the distribution graph of the variances of the principal components [scree plot] reaches a plateau after the first principal component) was used in calculating associations as a covariate (to exclude the factor of hidden stratification of the analyzed cohort, including those related to kinship);
(d) Adjustments for multiple comparisons; a permutation test with the calculation of the p_perm_ index was used to correct the number of studied SNPs (the permutation test effectively solves the problem of multiple comparisons even when analyzing GWAS data [92,93]); to correct the number of considered genetic models (n = 3), the Bonferroni amendment was additionally introduced, which was equal to 3 [79]; the association parameter corresponding to p_perm_ < 0.017 (0.05/3) was recognized as statistically significant;
(e) Mandatory assessment of the power (power_post hoc_) of the obtained associative relationship indicators (power_post hoc_ calculations were performed in the Quanto_v.1.2.4 program [94]).

### 4.4. Replication of Association of the rs10454142 PPP1R21/KLRAQ1 and Its Proxy SNPs with the PE Using GWAS Data

To confirm the association of rs10454142 *PPP1R21/KLRAQ1* with PE found in our study, a replicative study of the links of this polymorphism and SNPs in linkage disequilibrium (LD) with it (r^2^ > 0.20) with PE was conducted using GWAS data from the UK Biobank (http://geneatlas.roslin.ed.ac.uk/; accessed—17 August 2026) [95] and FinnGen database (https://public-metaresults-fg-ukbb.finngen.fi/about; date of access—17 August 2026) [96]. The list of proxy variants for rs10454142 *PPP1R21/KLRAQ1* was established using the LDlink (LDproxy) program (data from the “1000 Genomes” project (GRCh37)) among Europeans (Finnish in Finland (FIN)) were used (https://ldlink.nih.gov/ldproxy accessed 13 August 2026) [97]. For polymorphisms that were in LD with rs10454142 *PPP1R21/KLRAQ1* and showed associations with PE using GWAS data (UK Biobank and FinnGen database), an interactive heat map and a matrix of pairwise LD statistics (for parameters r^2^ and D′) were constructed in the LDlink (LDmatrix) program.

### 4.5. Predictive Functions of PE-Associated Loci

We analyzed the potential functionality of the PE-associated SHBG-related locus rs10454142 *PPP1R21/KLRAQ1* and 10 variants strongly linked with it (the value of LD was r^2^ ≥ 0.80) in four directions: (1) in the liver, an organ in which SHBG is predominantly synthesized [36]; (2) in organs/cell cultures targeted for PE, such as trophoblast, amnion, placenta, and uterus [1,5]; (3) in organs that are important for the pathophysiology of PE (brain [cortex, hypothalamus, pituitary gland], ovaries, adipose tissue, thyroid gland, adrenal glands, arteries, and blood) [2,3,4]; and (4) in the body as a whole.

The assumed functionality of the PE-correlated polymorphism rs10454142 *PPP1R21/KLRAQ1* and 10 proxy SNPs was estimated comprehensively (using an in silico approach and current bioinformatic databases [98,99]), taking into account their involvement in epigenetically significant processes (epiQTL [the location of SNPs in regions of open chromatin, potential promoters/enhancers/active promoters/active enhancers, and their localization at binding sites to transcription factors and regulatory proteins were evaluated]) (HaploReg base, accessed—8 October 2025 [83]), association with gene expression (eQTL) and alternative splicing (sQTL) (GTExproject, accessed—12 October 2025 [100]), genome methylation (mQTL) (QTLbase, accessed—15 October 2025 [101]), alternative polyadenylation (3′aQTL) (3′aQTL-atlas, accessed—19 October 2025 [102]), and common biological pathways (STRING base, accessed—26 November 2025 [103]). The use of the in silico approach and materials from modern bioinformatic databases (listed above) obtained as a result of large-scale international experimental studies in the field of functional genomics (for example, the HaploReg database used in this work integrates information from the ENCODE and Roadmap Epigenomics projects) makes it possible, without conducting independent experiments, to obtain reliable data on the potential functionality of the studied polymorphisms in various organs and tissues. These data suggest possible biological mechanisms underlying the identified associations.

## 5. Conclusions

The SHBG-related genetic variant, rs10454142 *PPP1R21/KLRAQ1*, was associated with PE. The revealed association of this locus with PE may be based on its alleged pronounced functionality (and its proxy variants) both in the liver (the main organ of SHBG formation) and in organs/cell cultures targeted for PE (trophoblast, amnion, placenta, and uterus), thus affecting the regulation of gene transcription, nucleic acid metabolism, and embryo development.

## Figures and Tables

**Table 1:** Phenotypic characteristics of the study participants.

Parameters	Preeclampsia, $\bar{X}$ ± SD/% (n)	Controls, $\bar{X}$ ± SD/% (n)	*p*
*N*	431	460	-
Age, years (min–max)	27.36 ± 5.06 (17–43)	26.58 ± 4.94 (16–42)	**0.02**
Pre-pregnancy BMI, kg/m^2^	24.85 ± 5.23	23.56 ± 3.61	**0.008**
Family history of preeclampsia	23.43 (101)	12.17 (56)	**0.0005**
Smoker (yes)	46.17 (199)	52.61 (242)	0.06
Alcohol consumption (yes)	75.64 (326)	79.35 (365)	0.21
Pre-pregnancy blood pressure (BP)			
Systolic BP, mm Hg	112.82 ± 9.85	111.23 ± 8.79	0.32
Diastolic BP, mm Hg	71.90 ± 7.31	72.26 ± 7.09	0.16
Mean BP, mm Hg	85.56 ± 7.86	85.23 ± 7.50	0.27
Pulse BP, mm Hg	39.92 ± 5.20	39.01 ± 3.69	0.14
The indicators of this pregnancy			
Gestational age at recruitment (mean, weeks) (min–max)	36.84 ± 3.32 (28–38)	38.72 ± 1.54 (37–40)	**<0.0001**
Gestational age at onset of PE (mean, weeks)	34.56 ± 3.67	-	-
Gestational age at onset of PE, early onset/late onset	14.15/85.85 (61/370)	-	-
Severity of PE, severe/mild	16.70/83.30 (72/359)	-	-
Fetal growth restriction	19.26 (83)	-	-
Age at menarche and menstrual cycle			
Age at menarche (mean, years)	12.45 ± 1.01	12.56 ± 1.02	0.62
Duration of bleeding menstrual (mean, days)	4.88 ± 0.96	5.00 ± 0.75	0.28
Menstrual cycle length (mean, days)	27.94 ± 2.93	28.39 ± 1.65	0.16
Reproductive status			
First pregnancy	39.44 (170)	42.39 (195)	0.40
No of gravidity (mean)	1.37 ± 1.70	1.08 ± 2.00	**0.04**
No of births (mean)	0.50 ± 0.71	0.53 ± 0.78	0.53
No of spontaneous abortions (mean)	0.21 ± 0.47	0.14 ± 0.32	**0.02**
No of induced abortions (mean)	0.62 ± 1.08	0.40 ± 0.72	**0.004**
No of stillbirths	0.04 ± 0.19	0.01 ± 0.06	**0.02**
Somatic pathologies			
Cardiovascular	13.69 (59)	9.78 (45)	0.09
Kidney	5.34 (23)	3.69 (17)	0.31
Endocrine	3.02 (13)	1.96 (9)	0.42
Gastrointestinal	2.32 (10)	2.83 (13)	0.79
Obesity	16.71 (72)	6.52 (30)	**0.0005**

Note: *p* values < 0.05 are shown in bold.

**Table 2:** Associations of the significant SHBG gene polymorphisms with preeclampsia.

SNP (Alleles: ref>alt) Gene	N	Additive Model				Dominant Model				Recessive Model			
		OR	95% CI		*p*	OR	95% CI		*p*	OR	95% CI		*p*
			L95	U95			L95	U95			L95	U95	
rs17496332 (A>G) *PRMT6*	865	1.03	0.85	1.25	0.782	1.01	0.77	1.34	0.919	1.08	0.74	1.58	0.686
rs780093 (C>T) *GCKR*	889	0.99	0.82	1.20	0.945	0.94	0.71	1.24	0.671	1.08	0.75	1.54	0.675
rs10454142 (T>C) *PPP1R21/KLRAQ1*	880	1.21	0.99	1.48	0.063	1.14	0.87	1.50	0.325	**1.73**	**1.16**	**2.66**	**0.015**
rs3779195 (T>A) *BAIAP2L1*	878	1.10	0.86	1.41	0.436	1.14	0.85	1.52	0.394	1.07	0.53	2.14	0.855
rs440837 (A>G) *ZBTB10*	869	1.02	0.81	1.29	0.866	1.00	0.76	1.31	0.996	1.18	0.61	2.29	0.621
rs7910927 (G>T) *JMJD1C*	883	0.84	0.70	1.02	0.082	0.82	0.61	1.11	0.200	0.78	0.57	1.07	0.119
rs4149056 (T>C) *SLCO1B1*	851	1.21	0.96	1.53	0.102	1.17	0.88	1.55	0.278	1.83	0.99	3.36	0.053
rs8023580 (T>C) *NR2F2-AS1*	882	1.21	0.99	1.48	0.069	1.29	0.99	1.69	0.063	1.24	0.79	1.95	0.354
rs12150660 (G>T) *SHBG*	888	0.77	0.57	1.05	0.096	0.73	0.51	1.04	0.084	0.89	0.49	1.61	0.696
rs727428 (C>T) *SHBG*	890	0.84	0.61	1.16	0.295	0.90	0.62	1.32	0.602	0.80	0.50	1.30	0.375
rs1641549 (C>T) *TP53*	886	1.01	0.75	1.37	0.942	1.20	0.85	1.71	0.306	0.59	0.31	1.11	0.099

Note: All results were obtained after adjustment for covariates; OR—odds ratio; 95% CI—95% confidence interval; *p* values < 0.05 are shown in bold.

**Table 3:** Replication of the association between proxy variants for the PE-associated SHBG-related variant rs10454142 *PPP1R21/KLRAQ1* and PE risk using UK Biobank and FinnGen GWAS data.

SNP	Position (GRCh37)	Effect Allele	Gestational Hypertension/ Preeclampsia (UK Biobank GWAS Data) Case-1829 Control-243665		Preeclampsia			
					FinnGen GWAS Data, Case-9256 Control-95490		Meta-Analysis of UK Biobank and FinnGen GWAS Data, Case-9465 Control-485003	
			OR	*p*	β	*p*	β	*p*
rs35839493	48593077	G	0.86	0.041	0.05	0.025	0.05	0.023
rs4953592	48776217	A	0.89	0.019				
rs79886465	48789709	C	1.13	0.020				
rs17037412	48809965	T	1.11	0.040				
rs7574549	48822540	C	1.20	0.008	0.05	0.045	0.05	0.043
rs1045919	48825109	G	1.19	0.011	0.04	0.050	0.04	0.048
rs11901451	48829030	G	1.19	0.010	0.04	0.050	0.04	0.047
rs6739536	48831901	A	1.13	0.047				
rs6752945	48831931	C	1.13	0.047				

Note: The table presents data at a significance level of *p* ≤ 0.05.

**Table 4:** Possible functionality of the PE-associated SHBG-related variant rs10454142 *PPP1R21/KLRAQ1* (highlighted in bold) and its proxy loci (r^2^ ≥ 0.80) (in silico information).

SNP (Position hg38) (r^2^, D′)	Liver—The Organ in Which SHBG Is Synthesized (HaploReg, GTE-Portal and QTLbase Data)	PE-Targeted Organs and Cell Cultures				In the Organism (In Total)				
		H1 BMP4 Derived Trophoblast Cultured Cells (HaploReg Data)	Placenta (HaploReg Data)	Placenta Amnion (HaploReg Data)	Uterus (GTE-Portal Data)	eQTL (GTE-Portal Data)	sQTL (GTE- Portal Data)	3′aQTL (3′aQTL- Atlas Data)	mQTL (QTLbase Data)	Transcription Factors (HaploReg Data)
rs17855177 (48375113) (r^2^ = 0.81, D′ = 0.99)	*GTF2A1L* (eQTL) *PPP1R21* (sQTL) *FOXN2* (mQTL)				*PPP1R21* (sQTL)	*MIR548BAHG*, *FOXN2*, *FSHR*, *GTF2A1L*, *LHCGR*, *MSH6*, *PPP1R21*, *PPP1R21-DT*, *STON1*, *STON1-GTF2A1L*	*FOXN2*, *PPP1R21*, *STON1*, *STON1-GTF2A1L*	*PPP1R21*	*FOXN2*, *PPP1R21*, *KLRAQ1*	
rs78597273 (48380665) (r^2^ = 0.81, D′ = 0.99)	*GTF2A1L* (eQTL) *PPP1R21* (sQTL) *FOXN2* (mQTL)				*PPP1R21* (sQTL)	*MIR548BAHG*, *FOXN2*, *FSHR*, *GTF2A1L*, *LHCGR*, *MSH6*, *PPP1R21*, *PPP1R21-DT*, *STON1*, *STON1-GTF2A1L*	*FOXN2*, *PPP1R21*, *STON1*, *STON1-GTF2A1L*	*PPP1R21*	*FOXN2*, *PPP1R21*, *KLRAQ1*	MIZF
rs11689645 (48381420) (r^2^ = 0.81, D′ = 0.99)	*GTF2A1L* (eQTL) *PPP1R21* (sQTL) *FOXN2* (mQTL)				*PPP1R21* (sQTL)	*MIR548BAHG*, *FOXN2*, *FSHR*, *GTF2A1L*, *LHCGR*, *MSH6*, *PPP1R21*, *PPP1R21-DT*, *STON1*, *STON1-GTF2A1L*	*FOXN2*, *PPP1R21*, *STON1*, *STON1-GTF2A1L*	*PPP1R21*	*FOXN2*, *PPP1R21*, *KLRAQ1*	AP-1, AP-2, BAF155, BATF, GR, Myc, BCL, Bach1, Bach2, GATA, HMGN3, KAP1, Maf, NF-E2, STAT, PRDM1, TCF4, p300
rs111960813 (48404376) (r^2^ = 0.80, D′ = 0.93)	*PPP1R21* (sQTL) *FOXN2* (mQTL)				*PPP1R21* (sQTL)	*MIR548BAHG*, *FOXN2*, *FSHR*, *GTF2A1L*, *LHCGR*, *MSH6*, *PPP1R21*, *PPP1R21-DT*, *STON1*, *STON1-GTF2A1L*	*FOXN2*, *PPP1R21*, *STON1*, *STON1-GTF2A1L*	*PPP1R21*	*FOXN2*, *PPP1R21*, *KLRAQ1*	ELF1, Myc, ZBRK1
rs56391806 (48404838) (r^2^ = 0.85, D′ = 0.98)	*PPP1R21* (sQTL) *FOXN2* (mQTL)				*PPP1R21* (sQTL)	*ELOBP3*, *MIR548BAHG*, *FOXN2*, *FSHR*, *GTF2A1L*, *LHCGR*, *MSH6*, *PPP1R21*, *PPP1R21-DT*, *STON1*, *STON1-GTF2A1L*	*FOXN2*, *PPP1R21*, *STON1*, *STON1-GTF2A1L*	*PPP1R21*	*FOXN2*, *PPP1R21*, *KLRAQ1*	Fox, Hoxb6
rs55744465 (48405316) (r^2^ = 0.85, D′ = 0.98)	*PPP1R21* (sQTL) *FOXN2* (mQTL)				*PPP1R21* (sQTL)	*MIR548BAHG*, *FOXN2*, *FSHR*, *GTF2A1L*, *LHCGR*, *MSH6*, *PPP1R21*, *PPP1R21-DT*, *STON1*, *STON1-GTF2A1L*	*FOXN2*, *PPP1R21*, *STON1*, *STON1-GTF2A1L*	*PPP1R21*	*FOXN2*, *PPP1R21*, *KLRAQ1*	Hoxa5
rs201414717 (48419259) (r^2^ = 1.00, D′ = 1.00)	* H3K4me1 H3K4me3 H3K27ac H3K9ac	H3K4me1	H3K4me1 H3K27ac	H3K27ac	*	*	*	*	*FOXN2*, *PPP1R21*, *KLRAQ1*	AP-4, CACD, WT1, YY1, TAL1, TCF12, Rad21, LBP-1, ZNF219
**rs10454142** **(48419260)**	***GTF2A1L*** ** (eQTL)** ***PPP1R21*** ** (sQTL)** ***FOXN2*** ** (mQTL)** **H3K4me1** **H3K4me3 H3K27ac H3K9ac**	**H3K4me1**	**H3K4me** **H3K27ac**	**H3K27ac**	***PPP1R21*** ** (sQTL)**	***MIR548BAHG*** **, ** ***FOXN2*** **, ** ***FSHR*** **, ** ***GTF2A1L*** **, ** ***LHCGR*** **, ** ***PPP1R21*** **, ** ***PPP1R21-DT*** **, ** ***STON1*** **, ** ***MSH6*** **, ** ***STON1-GTF2A1L***	***FOXN2*** **, ** ***PPP1R21*** **, ** ***STON1*** **, ** ***STON1-GTF2A1L***	***PPP1R21***	***FOXN2*** **, ** ***PPP1R21*** **, ** ***KLRAQ1***	**NF-kappaB**
rs10454143 (48419261) (r^2^ = 1.00, D′ = 1.00)	*GTF2A1L* (eQTL) *PPP1R21* (sQTL) *FOXN2* (mQTL) H3K4me1 H3K4me3 H3K27ac H3K9ac	H3K4me1	H3K4me1 H3K27ac	H3K27ac	*PPP1R21* (sQTL)	*MIR548BAHG*, *FOXN2*, *FSHR*, *GTF2A1L*, *LHCGR*, *MSH6*, *PPP1R21*, *PPP1R21-DT*, *STON1*, *STON1-GTF2A1L*	*FOXN2*, *PPP1R21*, *STON1*, *STON1-GTF2A1L*	*PPP1R21*	*FOXN2*, *PPP1R21*, *KLRAQ1*	Barx1, CEBPD, Hoxa3
rs13399936 (48426987) (r^2^ = 0.87, D′ = 0.96)	*GTF2A1L* (eQTL) *PPP1R21* (sQTL) *FOXN2* (mQTL)	H3K4me1			*PPP1R21 (sQTL)*	*MIR548BAHG*, *FOXN2*, *FSHR*, *GTF2A1L*, *LHCGR*, *MSH6*, *PPP1R21*, *PPP1R21-DT*, *STON1*, *STON1-GTF2A1L*	*FOXN2*, *PPP1R21*, *STON1*, *STON1-GTF2A1L*	*PPP1R21*	*FOXN2*, *PPP1R21*, *KLRAQ1*	
rs4638844 (48427445) (r^2^ = 0.81, D′ = 0.94)	* FOXN2 (mQTL)	H3K4me1			*	*	*	*	*FOXN2*, *PPP1R21*, *KLRAQ1*	CIZ, FAC1, Foxa, Foxd3, Foxj2, Foxo, Foxk1, Foxp1, HDAC2, Irf, Pax-4, Sox, RREB-1, Zfp105, p300

Note: *—The information in the GTE-portal and 3′aQTL-atlas databases is not provided; H3K4me1—SNP location in the region of H3K4me1 histones marking enhancers; H3K27ac—active enhancers; H3K4me3—promoters; H3K9ac—active promoters; bold highlights PE-linked SNP.

**Table 5:** GWAS data on associations of the studied candidate gene polymorphisms with the circulating SHBG and other sex hormone concentrations.

SNP, Gene	Chromosome Position (hg38)	Phenotype	Association (Significance) (Effect Allele)	Reference
rs17496332 *PRMT6*	1p13.3 (107003753)	SHBG	β = −0.028 (*p* = 1 × 10^−11^) (A)	[40]
rs780093 *GCKR*	2p23.3 (27519736)	SHBG	β = −0.032 (*p* = 2 × 10^−16^) (T)	[40]
rs10454142 *PPP1R21/* *KLRAQ1*	2p16.3(48419260)	SHBG	β = 0.026 (*p* = 1 × 10^−7^) (T)	[40]
rs3779195 *BAIAP2L1*	7q21.3 (98364050)	SHBG	β = −0.033 (*p* = 3 × 10^−8^) (A)	[40]
		SHBG (pre-menopausal women)	β = −2.41 (*p* = 9 × 10^−9^) (A)	[45]
rs440837 *ZBTB10*	8q21.13 (80549739)	SHBG	β = −0.030 (*p* = 3 × 10^−9^) (A)	[40]
		SHBG (post-menopausal women)	β = 1.43 (*p* = 1 × 10^−12^) (G)	[45]
rs7910927 *JMJD1C*	10q21.3 (63379150)	SHBG	β = −0.048 (*p* = 6 × 10^−35^) (T)	[40]
rs4149056 *SLCO1B1*	12p12.1 (21178615)	SHBG	β = 0.029 (*p* = 2 × 10^−8^) (T)	[40]
		Total testosterone	β = 0.028 (*p* = 5 × 10^−10^) (C)	[50]
		SHBG	β = −0.065 (*p* = 5 × 10^−48^) (C)	[50]
		SHBG (pre-menopausal women)	β = −0.062 (*p* = 8 × 10^−11^) (C)	[50]
		SHBG (post-menopausal women)	β = −0.079 (*p* = 7 × 10^−34^) (C)	[50]
		Free testosterone	β = 0.02 (*p* = 2 × 10^−16^) (C)	[45]
		SHBG	β = 0.030 (*p* = 1 × 10^−73^) (T)	[43]
		Total testosterone	β = −0.029 (*p* = 1 × 10^−14^) (T)	[43]
		Free testosterone	β = −0.043 (*p* = 3 × 10^−35^) (T)	[43]
rs8023580 *NR2F2-AS1*	15q26.2 (96165062)	SHBG	β = −0.03 (*p* = 8 × 10^−12^) (T)	[40]
rs12150660 *SHBG*	17p13.1 (7618597)	SHBG	β = 0.103 (*p* = 2 × 10^−106^) (T)	[40]
		SHBG	β = 6.14 (*p* = 1 × 10^−300^) (T)	[45]
rs727428 *SHBG*	17p13.1(7634474)	SHBG	β = −0.126 (*p* = 2.09 × 10^−16^) (T)	[41]
		Free testosterone	β = 0.095 (*p* = 8.3 × 10^−309^) (T)	[43]
		Estradiol	β = −0.041 (*p* = 2.10 × 10^−10^) (T)	[50]
		Free testosterone	β = −0.001 (*p* = 5.00 × 10^−21^) (C)	[45]
		SHBG (pre-menopausal women)	β = 7.25 (*p* = 5.43 × 10^−109^) (C)	[45]
		Free testosterone (post-menopausal women)	β = −0.019 (*p* = 3.09 × 10^−25^) (C)	[45]
		Free testosterone	β = −0.021 (*p* = 1.03 × 10^−48^) (C)	[45]
rs1641549 *SHBG*	17p13.1 (7671457)	SHBG	β = −0.127 (*p* = 1.21 × 10^−15^) (T)	[42]

## Data Availability

The original contributions presented in this study are included in the article/supplementary material. Further inquiries can be directed to the corresponding author.

## Supplementary Materials

The following supporting information can be downloaded at: https://www.mdpi.com/article/10.3390/ijms27188387/s1.

## Abbreviations

The following abbreviations are used in this manuscript:

PE	Preeclampsia
SNP	Single nucleotide polymorphism
GWAS	Genome-wide association studies
SHBG	Sex hormone-binding globulin
LD	Linkage disequilibrium
