# Supplementary material for: Polymorphic Variant Associated with Sex Hormone-Binding Globulin Level Is a Risk Factor for Preeclampsia
Source: Int J Mol Sci. 2026 Sep 20;27(18):8387. doi: 10.3390/ijms27188387 (PMC13607597; doi:10.3390/ijms27188387)
Supplement: Supplementary file 1 [file ijms-27-08387-s001.zip › Suppl Table S2.pdf]

Supplementary Table S2. The genotype counts and allele frequencies of the studied SNPs in the preeclampsia and control groups

Chr	SNP	Gene	Minor allele	Major allele	Minor allele frequency	Number of the studied chromosomes	Genotype distribution*	H_o	H_e	P_{HWE}
Preeclampsia (n=431)										
1	rs17496332	*PRMT6*	G	A	0.374	816	63/179/166	0.439	0.468	0.205
2	rs780093	*GCKR*	T	C	0.399	860	73/197/160	0.458	0.480	0.366
2	rs10454142	*PPP1R21/KLRAQ1*	C	T	0.337	848	54/178/192	0.420	0.447	0.232
7	rs3779195	*BAIAP2L1*	A	T	0.180	848	17/119/288	0.281	0.296	0.323
8	rs440837	*ZBTB10*	G	A	0.233	834	20/154/243	0.369	0.357	0.583
10	rs7910927	*JMJD1C*	T	G	0.464	862	91/218/122	0.506	0.497	0.772
12	rs4149056	*SLCO1B1*	C	T	0.240	794	28/135/234	0.340	0.365	0.208
15	rs8023580	*NR2F2-AS1*	C	T	0.308	856	44/176/208	0.411	0.427	0.496
17	rs12150660	*SHBG*	T	G	0.230	860	28/142/260	0.330	0.355	0.173
17	rs727428	*SHBG*	T	C	0.381	862	58/212/161	0.492	0.471	0.414
17	rs1641549	*TP53*	T	C	0.243	856	22/164/242	0.383	0.368	0.433
Control (n=460)										
1	rs17496332	*PRMT6*	G	A	0.368	916	66/205/187	0.448	0.465	0.422
2	rs780093	*GCKR*	T	C	0.404	920	75/222/163	0.483	0.482	1.000
2	rs10454142	*PPP1R21/KLRAQ1*	C	T	0.304	914	39/200/218	0.438	0.423	0.509
7	rs3779195	*BAIAP2L1*	A	T	0.171	910	18/120/317	0.264	0.284	0.136
8	rs440837	*ZBTB10*	G	A	0.231	906	18/173/262	0.382	0.355	0.144
10	rs7910927	*JMJD1C*	T	G	0.508	906	118/224/111	0.495	0.500	0.851
12	rs4149056	*SLCO1B1*	C	T	0.209	908	19/152/283	0.335	0.331	0.888
15	rs8023580	*NR2F2-AS1*	C	T	0.277	910	42/168/245	0.369	0.401	0.101
17	rs12150660	*SHBG*	T	G	0.256	918	34/167/258	0.364	0.381	0.329
17	rs727428	*SHBG*	T	C	0.386	920	67/221/172	0.480	0.474	0.844
17	rs1641549	*TP53*	T	C	0.236	918	34/149/276	0.325	0.361	0.038

Note: * minor allele homozygotes / heterozygotes / major allele homozygotes
